# Roles and potential therapeutic targets of the ubiquitin proteasome system in muscle wasting

**DOI:** 10.1186/1471-2091-8-S1-S7

**Published:** 2007-11-22

**Authors:** David Nury, Christine Doucet, Olivier Coux

**Affiliations:** 1 CRBM-CNRS UMR5237, IFR22, 1919 route de Mende, 34000 Montpellier, France; 2 Molecular and Cell Biology Laboratory, Salk Institute, 10010 North Torrey Pines Road, CA 92037-1099 La Jolla, USA; 3 CRBM-CNRS, 1919 route de Mende, 34293 Montpellier cedex 5, France

## Abstract

Muscle wasting, characterized by the loss of protein mass in myofibers, is in most cases largely due to the activation of intracellular protein degradation by the ubiquitin proteasome system (UPS). During the last decade, mechanisms contributing to this activation have been unraveled and key mediators of this process identified. Even though much remains to be understood, the available information already suggests screens for new compounds inhibiting these mechanisms and highlights the potential for pharmaceutical drugs able to treat muscle wasting when it becomes deleterious. This review presents an overview of the main pathways contributing to UPS activation in muscle and describes the present state of efforts made to develop new strategies aimed at blocking or slowing muscle wasting.

**Publication history: **Republished from Current BioData's Targeted Proteins database (TPdb; ).

## Roles of the UPS in muscle wasting

Skeletal muscle wasting, or atrophy, is characterized by a reduction in muscle mass due to an imbalance between protein synthesis and degradation, resulting in a loss of net protein content in the myofibers (see reviews cited in [1] for more information). Even though there is clear evidence that a reduction in protein synthesis participates in this process, there is now a large body of experiments showing that a central mechanism in muscle wasting is increased intracellular proteolysis, due in particular (but not exclusively [2]) to the activation of the ubiquitin proteasome system (UPS) [3,4].

Muscle wasting occurs in a variety of physiological or pathological circumstances, including inactivity or muscle disuse, fasting and many diseases such as cancer, renal failure or trauma [5]. As skeletal muscle is the major protein reservoir of the body, muscle wasting has beneficial effects in its initial stage since it provides the organism with an ample pool of free amino acids that can be reused for energy production or protein synthesis in vital organs [6]. However, in a variety of pathologies such as cancer, sepsis and diabetes, this catabolic state is maintained over a long period of time and can become life-threatening; this condition is often referred to as cachexia [7].

The aim of this review is to provide an overview on the role of the UPS in muscle wasting. For the sake of space, only the mechanisms presently thought to be common to most types of muscle wasting are described, and the reader is invited to refer to numerous recent reviews for more detailed information on specific topics [1,5,6,8-11]. It should be noted that most of the results described in this review were obtained with animal models of severe muscle wasting. Therefore, extrapolation to humans of the mechanisms presented below should be treated with caution.

### UPS components involved in muscle wasting

Initial studies, mostly performed using muscles isolated from animals subjected to induced muscle wasting, led to the conclusion in the early 1990s that increased ATP-dependent proteolysis accounted for a large part of muscle protein loss in several muscle wasting models [12]. After the subsequent recognition that the UPS (initially discovered and dissected in rabbit reticulocyte lysate [13-15]) was responsible for ATP-dependent proteolysis in mammalian cells [16], and the demonstration that its general inhibition by proteasome inhibitors reduces the accelerated proteolysis in atrophying muscles [17], several groups turned to the analysis of expression of individual UPS components in different experimental models of muscle wasting (see Table 1).

**Table 1:** Main conditions used to induce muscle wasting in animals (mice and rats) and the variation of key components involved in UPS activation. Please note that the models in this table were generated only for the experiments: muscle wasting was induced in normal animals by various treatments and the molecular mechanisms subsequently analyzed.

**Main models of muscle wasting**	**Variation of key components involved in UPS activation**					
	
	**UPS mRNAs**	**N-end rule pathway**	**NFκB activity**	**MuRF-1**	**MAFbx**	**Selected reference**
**Cancer cachexia**						[84,100]
**Sepsis**						[101,102]
**Hindlimb suspension**						[89,103]
**Denervation**		?				[17,31,104]
**Hyperthyroidism**		?				[17,105]
**Burn injuries**						[106,107]
**Cirrhosis**		?				[108,109]
**Starvation**						[110,111]
**Diabetes**						[56,105,112]
**Uremia**		?				[37,113]

: upregulation; ?: not determined or unclear.

In general, the UPS functions in two distinct steps to degrade intracellular proteins. First, the substrate protein is labeled by covalent linkage of polyubiquitin (polyUb) chain(s) to one or several lysine residue(s) by the action of an enzymatic cascade involving three types of factors, referred to as E1, E2s and E3s [18]. In this reaction, the E3s act as substrate recognition factors and are thus the main determinants of the specificity of the UPS [19]. Consequently, their number is very high, with a rough estimation of several hundred E3s present in the genome, depending on the organism [20]. The second step in protein degradation by the UPS is the degradation of the polyubiquitylated substrate by the 26S proteasome, a large compartmentalized protease comprising the 20S proteasome, which contains the proteolytic active sites in its internal catalytic chamber, and the 19S regulatory complex, which is essential for degradation of ubiquitylated proteins [21,22].

A common theme in most models of muscle wasting is a significant increase at the mRNA level of many components of the UPS. These include ubiquitin (Ub), E2s, proteasome subunits (20S and 19S), deubiquitylating enzymes and certain E3s [6,8,23-25]. The accepted interpretation is that this upregulation of UPS mRNAs participates in the overall activation of the UPS observed during muscle wasting, in line with the fact that an increase in (i) the rate of Ub conjugation to proteins, (ii) the intracellular level of ubiquitylated proteins and (iii) proteasome activity has been described in most muscle wasting conditions [6,8]; see also Table 1]. However, it should be noted that, despite the upregulation of their corresponding mRNA, the level of many UPS proteins does not appear significantly altered during muscle wasting. The reasons for this discrepancy are unclear, but an interesting hypothesis is that the increased synthesis of these proteins is compensated by an accelerated turnover under the conditions analyzed.

The first specific UPS pathway described to be involved in muscle wasting was the ‘N-end rule’ pathway ([26,27]; see also section on *Substrates of the UPS during muscle wasting*), which rapidly degrades proteins with destabilizing N-terminal residues (see [28] for review). In mammals, this pathway involves a specific E2 (E2-14K) and several E3 isoforms [29], among which E3α-II (also known as UBR2) is increased in skeletal muscle during cachexia [27]. The specific inhibition of this E3 reduces the rate of ubiquitylation of soluble or model proteins in muscle extracts from tumor-bearing or septic rats, as well as in C2C12 cell extracts [26,27].

Transcriptome analyses showed that two other types of E3 or E3 components are upregulated in most muscle wasting models. These are the F-box component MAFbx (also known as Atrogin-1) of the SCF^MAFbx^ E3 and the MuRF family of RING finger E3s [30,31], which interact with and possibly ubiquitylate a variety of myofibrillar proteins [32,33]. Expression of MAFbx and MuRF-1 mRNAs increases dramatically in catabolic states and mice deficient in either of them are partially resistant to denervation atrophy [31]. Therefore, MAFbx and MuRF-1 play a crucial role in the loss of muscle proteins and their mRNAs are now considered to be specific atrophic markers.

### Substrates of the UPS during muscle wasting

The major proteins in muscle are the myofibrillar components; however, although monomeric actin and myosin can be degraded by the UPS, it is assumed that this is not the case in intact myofibers since specific interactions between these proteins protects them from degradation by this system [34]. Thus, an early step in muscle wasting must be the dissociation or at least the destabilization of the myofibrils, a process in which non-UPS proteases shown to be involved in muscle wasting (such as calpains [35,36], caspase 3 [37] or cathepsin L [38]) probably play a key role. Though the early involvement of non-UPS proteases in the destabilization of myofibrils has not been formally demonstrated, this hypothesis is likely since it helps to explain the observed role of the UPS N-end rule pathway in muscle wasting. Indeed, this pathway requires the substrate to expose a destabilizing N-terminal residue [28], a feature that is rare for native cellular proteins; however, polypeptides with a destabilizing N-terminal residue can be produced by proteolytic cleavage of native proteins by cellular proteases [39].

Another mechanism that controls myofibril organization involves the sarcomeric scaffold protein titin, which possesses a protein kinase domain essential for sarcomere integrity [40,41]. Interestingly, the activity of this domain is modulated by mechanical strain [42], allowing titin to convert tension forces into molecular signals. This function controls both the intracellular level and localization of the RING finger E3 MuRF-2 (a protein closely related to MuRF-1), which is associated with titin in the sarcomeres through interaction with the UBD (Ub binding domain [43])-containing proteins Nbr1 and p62, and is translocated to the nucleus upon muscle inactivity [44]. Nuclear localization of MuRF-2 in turn leads to nuclear exclusion of SRF (serum response factor) [44], a transcription factor central to muscle response to hypertrophic stimuli. Thus, titin appears to be an important relay in the control of muscle disuse atrophy, since it is able to couple mechanical inactivity with muscle wasting by triggering macromolecular rearrangements of sarcomeres and altering muscle transcriptional programs. The importance of the titin–Nbr1–p62–MuRF-2 interaction is underlined by a human mutation disrupting binding of Nbr1 to titin, which causes an autosomal dominant muscle disease called heredity myopathy with early respiratory failure (HMERF) [44]. In muscle biopsies of HMERF patients, the structure of sarcomeres is altered and the localization of Nbr1, p62 and MuRF-2 is abnormal [44].

In addition to these mechanisms, the crucial roles of MAFbx and MuRF-1 during atrophy indicate that these E3s target key negative regulators of muscle wasting for degradation. However, only a few actual or potential substrates have been identified for these E3s and the relevance of these substrates for muscle wasting remains unclear (see *Signaling pathways involved in muscle wasting)*.

### Signaling pathways involved in muscle wasting

Glucocorticoids are known to be important mediators of muscle wasting in various catabolic conditions [5]; indeed, dexamethasone triggers upregulation of various UPS components [45]. However, the underlying mechanisms of glucocorticoid signaling in muscle wasting are not yet fully understood [23].

Presently, two families of transcription factors have been clearly identified as crucial positive mediators of muscle wasting: the FoxO (forkhead box O) transcription factors and NFκB [9]. Of note, these factors are involved in the control of expression of the E3s MuRF-1 and MAFbx [46-48].

PI3K is activated by anabolic signals triggered by insulin or IGF-1 upon binding to their receptors, which in turn activates Akt [49] (a kinase central to muscle hypertrophy), as shown in transgenic mice in which an active form of Akt can be inducibly expressed in adult skeletal muscle [50]. Besides its function in stimulating protein synthesis via mTOR and GSK3 [9], a crucial role for activated Akt is to phosphorylate FoxO [46,47,51], leading to transcriptional inactivation of FoxO due to its release from DNA and 14-3-3 protein-mediated cytoplasmic sequestration (see [52] for review), and to subsequent inhibition of muscle wasting. In many systemic diseases, including diabetes, cancer and renal failure, low circulating levels of insulin and IGF-1, or resistance to their action, contribute to muscle wasting via inactivation of the PI3K/Akt pathway, which leads to the subsequent dephosphorylation and activation of FoxO [53-56]. Interestingly, it has recently been shown that myostatin treatment can also trigger the same effect in mice [57]. Myostatin is a TGFβ super-family member that acts as a negative regulator of muscle growth and is implicated in several forms of muscle wasting [58]. Myostatin signaling reverses the IGF-1/PI3K/Akt pathway by inhibiting phosphorylation of Akt, leading to activation of FoxO and thereby activation of E2-14K, MuRF-1 and MAFbx [57].

In many disease states, stress or inflammatory cytokines such as tumor necrosis factor alpha (TNFα) are produced [59]. Stimulation of their receptors activates the IKK kinase, which in turn phosphorylates the NFκB inhibitor IκBα, promoting its degradation by the UPS. This leads to the release of NFκB, which migrates to the nucleus to activate its target genes [60]. Demonstration of a role for this pathway in muscle wasting comes from many observations, including the fact that constitutive activation of IKK in transgenic mice triggered both a decrease in total muscle mass and an increase in atrophy markers, which were reversed by expression of IκBα [48]. A further observation supporting a role for this pathway in muscle wasting is that direct inhibition of NFκB inhibited cachexia in a mouse tumor model [61]. In addition, in cancer cachexia, tumor-derived factors such as proteolysis-inducing factor (PIF) [62,63], or elevated levels of endogenous factors such as angiotensin I and II, are thought to contribute to muscle atrophy through activation of the NFκB pathway. Supporting evidence comes from the fact that these factors induce increased expression of UPS components (proteasome subunits, E2-14K) and stimulate protein degradation in murine C2C12 myotubes in a manner requiring IκBα degradation and nuclear translocation of NFκB [64,65]. Interestingly, although activation of NFκB is also required for muscle disuse atrophy, the NFκB family members that are activated appear distinct from those activated by the TNFα pathway [1,66].

NFκB and FoxO are thus key players in the activation of muscle wasting. Interestingly, their known targets include MuRF-1 and MAFbx for FoxO [46,47], and MuRF-1 for NFκB [48]. However, as mentioned in the section on *Substrates of the UPS during muscle wasting*, the key substrates of these E3s in muscle wasting are unclear. Nevertheless, one hypothesis is that induction of MuRF-1 activity is important for the destabilization of myofibrillar complexes required for the subsequent degradation of myofibrillar proteins. Indeed, the interaction between MuRF-1 and titin has been shown to be important for sarcomere stability [67]. Furthermore, in yeast two-hybrid screens, MuRF-1 interacts with several myofibrillar proteins (titin, nebulin, the nebulin-related protein NRAP, troponin-I, troponin-T, myosin light chain 2, myotilin and T-cap) [33], suggesting that this E3 can mediate their ubiquitylation and degradation in muscle. MuRF-1-mediated ubiquitylation and degradation, however, has only been proven for troponin I [68] and possibly for myosin light chain 2 [33] in cardiac tissue. The lack of clear results regarding the role of MuRF-1 in the degradation of its other interactors could be due to its functional redundancy with MuRF-2.

The known targets of MAFbx (calcineurin A [32] and MyoD [69])) are unlikely to be crucial for muscle wasting, since calcineurin A does not appear to affect myofiber size [70] and MyoD is involved in muscle cell differentiation. The key substrates of MAFbx in muscle wasting thus remain to be identified. Interestingly, however, TNFα/NFκB signaling reduces both the level of MyoD mRNA [48] and the stability of MyoD [71], and forced expression of MAFbx inhibits the formation of myotubes in an *in vitro* model of muscle differentiation [69]. Thus, the role of MAFbx in the down-regulation of MyoD could in fact be indirectly important for muscle wasting, since the inactivation of MyoD functions necessary for the differentiation of muscle satellite cells possibly inhibits muscle regeneration.

### Concluding remarks

In this brief overview, we have summarized the role of the UPS as an essential mediator of muscle wasting. Even though much remains to be understood before a complete picture of the intricate mechanisms controlling muscle atrophy can emerge, our knowledge to date already suffices to demonstrate that the UPS is an attractive potential target for therapeutic treatments aimed at slowing down muscle atrophy when it becomes harmful to the organism [5]. The different levels of possible therapeutic intervention are discussed in the section on *Potential targets and drugs for muscle wasting therapy*.

## Analysis of muscle wasting: models, knockouts and assays

### Main models of muscle wasting

Muscle wasting occurs when the overall rate of protein proteolysis exceeds in a sustained manner that of protein synthesis in muscle. Since a variety of physiological and pathological conditions can influence the equilibrium between these two processes, several animal (mostly murine) models have been developed (see Table 1) to analyze the mechanisms of muscle mass loss in these different conditions [72]. For example, cancer, sepsis and cirrhosis are inflammatory conditions that induce muscle wasting through the inhibition of anabolic pathways and the secretion of cytokines (IL1, TNFα) or catabolic factors (PIF), while hindlimb suspension, denervation and sarcopenia are conditions in which disuse atrophy is present. In most models, despite variations in the extent of involvement of the UPS relative to other processes (including other proteolytic systems), activation of UPS-dependent proteolysis transpires to be a crucial event [6]. Moreover, as described in the section on *Roles of the UPS in muscle wasting*, biochemical and molecular analyses have determined that a convergent mechanism for this activation is the upregulation of crucial ubiquitylation pathways involving the N-End rule E3s as well as the E3s SCF^MAFbx^ and MuRF-1 [73], even though the substrates of these enzymes during muscle wasting remain unclear at present.

### Knockouts of components involved in muscle wasting

Several mouse strains deficient in components involved in muscle wasting have been constructed and tested for their protection against muscle atrophy (see Table 2). Manipulation of the N-end rule pathway, by deletion of the genes encoding either E2-14K or the E3 UBR1 [74,75], did not significantly impair muscle wasting, probably because of the functional redundancy between the many components of this pathway [29] and/or because this pathway is only responsible for the breakdown of soluble muscle proteins. By contrast, mice in which either the MAFbx- or MuRF-1-encoding gene has been deleted become partially resistant to atrophy induced by denervation, highlighting the crucial role of these two E3s in the process of muscle wasting. Finally, mice with altered control of the NFκB pathway demonstrate the key function of this pathway in inducing muscle atrophy.

**Table 2:** Mouse models genetically modified for proteins involved in muscle wasting

Genetically modified mice	Effect on muscle (muscle wasting condition tested)	Selected references
**KO MuRF-1**	Partial protection against atrophy (denervation)	[31]
**KO Atrogin-1/MAFbx**	Partial protection against atrophy (denervation)	[31]
**KO E2-14K**	Non-resistant to atrophy (fasting)	[74,114]
**KO UBR1**	Decreased muscle mass	[75]
**Mdx**	Model for Duchenne muscular dystrophy: in these mice blockade of NFκB partially restores muscle function	[115]
**KI FoxO**	FoxO overexpressing mice: negative effect on skeletal muscle mass	[116]
**MIKK constitutively active IKK**	Severe atrophy resembling cachexia	[48]
**MISR non-degradable IκB**	Protection against atrophy (denervation, tumor)	[48]

### Assays for analysis of muscle wasting

In addition to the measurement of muscle mass after isolation of specific muscles from model animals, protein breakdown in incubated muscle is often quantified by the release of tyrosine, which is neither synthesized nor metabolized in skeletal muscle [76]. Furthermore, release of 3-methylhistidine, a modified amino acid found in actin and myosin that can be neither reincorporated into protein nor metabolized, can also be measured [77,78].

## Potential targets and drugs for muscle wasting therapy

**Figure 1:**
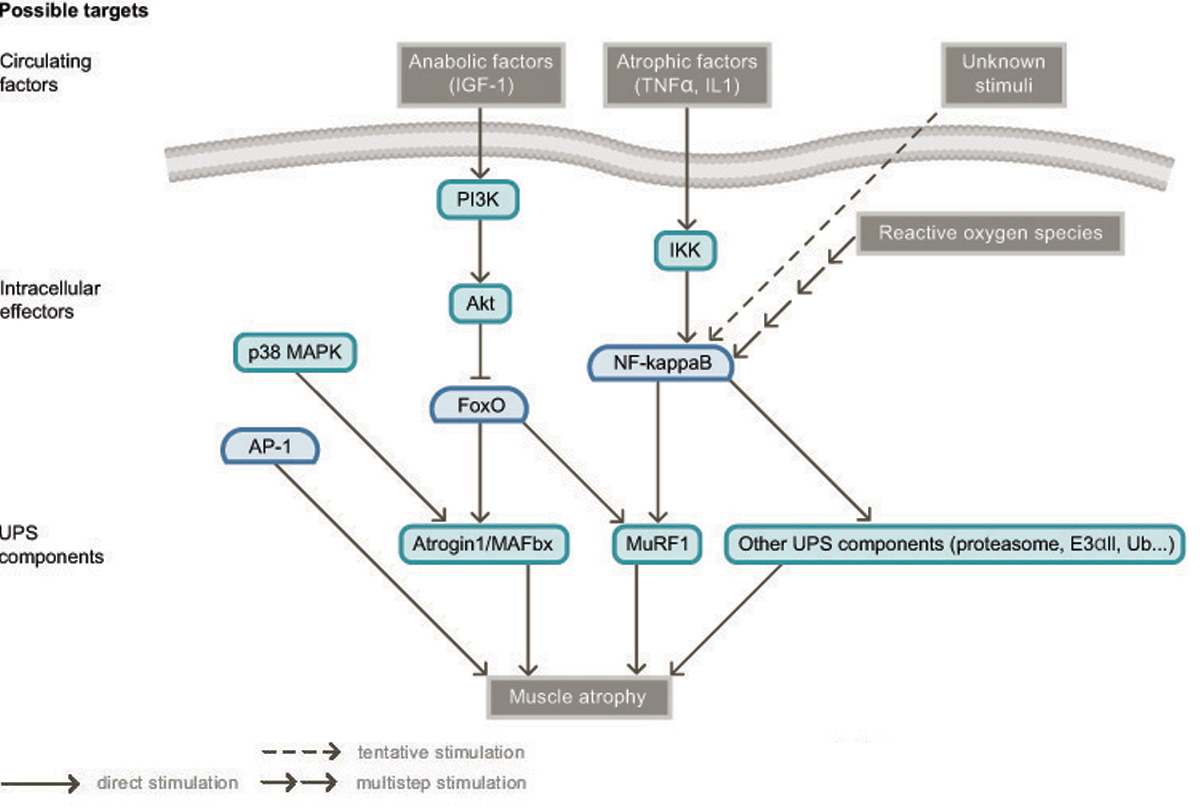
**Potential therapeutic targets in muscle wasting**. The figure illustrates possible therapeutic targets in muscle wasting, including circulating factors, intracellular effectors and components of the ubiquitin proteasome system (UPS).

As summarized in the section on *Roles of the UPS in muscle wasting* and figure 1, there are several levels of possible therapeutic intervention to limit muscle wasting. The first level is to modulate the expression or activity of circulating factors that either promote muscle hypertrophy (such as insulin or IGF-1) [79], inhibit muscle growth (such as myostatin) [58], or induce the UPS and/or NFκB (such as the cytokines TNFα or IL6, the secreted glycoprotein PIF, or oxidative stress) [5]. The second level involves targeting intracellular effectors of muscle wasting, such as the transcription factor FoxO or the NFκB pathway. Finally, a third level of possible therapeutic intervention is direct inhibition of the UPS.

As the exploration of these different possibilities is currently underway, only partial and limited information is presently available. To date, most compounds presently tested on animal models or in clinic for their ability to limit muscle wasting are commercially available chemicals, and many of them have been identified through empirical approaches aimed at treating cachexia [80]. Among those, EPA (eicosapentaenoic acid), which has been shown to down-regulate the increase in UPS activity seen in cachexia [81], and thalidomide, which inhibits production of TNFα by increasing the rate of degradation of its mRNA [82], have demonstrated a degree of efficacy [80,83,84].

Other inhibitors of TNFα expression represent attractive therapeutic strategies against cachexia. Although the xanthine derivative pentoxifylline [85] can suppress cachexia-induced activation of the UPS in rats [86], its effects in clinical trials have been uncertain [87]. However, another xanthine derivative, torbafylline (HWA 448), showed encouraging properties in a study on animal models, since it appeared more potent in preventing muscle wasting than pentoxifylline [88].

Several studies have analyzed the potential effect on muscle wasting of beta2 agonists such as clenbuterol [89], fenoterol [90] and formoterol [91]. These anabolic drugs appear effective against atrophy, mostly by counteracting activation of the UPS. Though the potential use of clenbuterol to treat muscle wasting appears limited since it has deleterious side effects in humans (particularly tachycardia and hypokalemia [92]), formoterol is already in use in human therapy (against chronic obstructive pulmonary disease) and thus appears to be an interesting candidate for human trials.

Due to the prominent role of NFκB in cachexia, drugs inhibiting the activation of this transcription factor have also been tested in various settings. Active molecules include HMB (β-hydroxy-β-methylbutyrate, a leucine metabolite) [80], ibuprofen [80] and resveratrol, the latter of which appears to be the most promising drug candidate for clinical use against muscle wasting [84,93].

Since the proteasome is a central component in muscle wasting, it represents an attractive target despite its pleiotropic functions. Indeed, many studies in animals have demonstrated that proteasome inhibitors are efficient at blocking muscle wasting [6,94]; however, no report on their effect on muscle wasting in humans has yet been published. The recent success of Millennium Pharmaceuticals with the proteasome inhibitor bortezomib (PS-341, Velcade®) for treatment of multiple myeloma [95] prompted many biotech or pharmaceutical companies to develop new molecules targeting the proteasome, including Nereus (NPI-0052), Proteolix (PR-171), Santhera and ALSTDF. Although muscle wasting is not the primary interest of these companies, these efforts could help to produce new compounds with interesting anti-atrophying properties in the future.

Finally, it is important to highlight that, when possible, muscular exercise is an efficient method of limiting disuse atrophy since, as shown by analyses in humans, it negatively influences the signaling pathways activated in muscle wasting, leading in particular to down-regulation of the E3s MAFbx and MuRF-1 [96].

## New frontiers in drug discovery

Knowledge of the molecular mechanisms responsible for muscle wasting, as summarized in figure 1, has been acquired relatively recently and much remains to be understood before a more accurate description of this process is obtained. For example, the contribution of the AP-1 transcription factors to muscle wasting [97], or of the p38 MAP kinase to activation of MAFbx [98], remains to be clarified. In addition, future studies will certainly characterize novel factors involved in atrophy and provide a better evaluation of the therapeutic potential of activating or inhibiting the many pathways controlling muscle wasting. Nevertheless, as several levels of therapeutic intervention have been identified and drug candidates are currently being tested, it is most likely that efficient drugs against muscle wasting will be soon available. However, the many problems of bioavailability and of specific targeting of muscle tissues remain to be solved in most cases.

A clear limitation in the current attempts to reduce muscle wasting is that the targeted molecules are not muscle-specific; meaning that alteration of their expression or activity is bound to induce side effects that could worsen the clinical status of the patient. A combination of drugs displaying synergistic effects, as shown recently for pentoxifylline and formoterol [55], could help to improve treatment since this strategy could allow use of sub-therapeutic doses of each drug, decreasing the possibility of harmful side effects.

Clearly, an alternative goal is to develop compounds that will target specific factors that are key for muscle wasting and that have no general functions outside muscle. In this respect, the E3s specifically induced during muscle wasting, such as the N-end rule E3s or MuRF-1, are extremely attractive candidates. However, as illustrated in the Mdm2/p53 pathway (see [99] for review), the best approach to obtain specific and efficient inhibitors of E3s is to aim to disrupt the interaction of the E3 with its substrate(s). This requires substantial information on the nature of the relevant substrates of the E3 and on the structure of the E3–substrate pair to be targeted. As such information is presently not available for the E3s involved in muscle wasting, important goals for the future are to identify their substrates and to define the structural basis of their interaction with these substrates.

## List of abbreviations

Akt = serine/threonine protein kinase; FoxO = forkhead box O; GSK3 = glycogen synthase kinase 3; IGF-1 = insulin-like growth factor 1; KI = knockin; KO = knockout; MAFbx = muscle atrophy F-box; MAP kinase = mitogen-activated protein kinase; mTOR = mammalian target of rapamycin; MuRF1 = muscle RING finger 1; NFκB = nuclear factor kappa B; PI3K = phosphatidylinositol 3-kinase; PIF = proteolysis inducing factor; SCF = SKP1–cullin–F-box protein; SRF = serum response factor; TGFβ = transforming growth factor beta; TNFα = tumor necrosis factor alpha; Ub = ubiquitin; UBD = ubiquitin binding domain; UPS = ubiquitin proteasome system.

## Competing interests

The authors declare that they have no competing interests.

## Publication history

Republished from Current BioData's Targeted Proteins database (TPdb; ).
